# Spectroscopic On-Line Monitoring of Cu/W Contacts Erosion in HVCBs Using Optical-Fibre Based Sensor and Chromatic Methodology

**DOI:** 10.3390/s17030519

**Published:** 2017-03-06

**Authors:** Zhixiang Wang, Gordon R. Jones, Joseph W. Spencer, Xiaohua Wang, Mingzhe Rong

**Affiliations:** 1 State Key Laboratory of Electrical Insulation for Power Equipment, School of Electrical Engineering, Xi’an Jiaotong University, 28 Xianning West Rd, Xi’an 710049, China; wangzhixiang.215@gmail.com (Z.W.); xhw@mail.xjtu.edu.cn (X.W.); 2 Department of Electrical Engineering & Electronics, University of Liverpool, Brownlow Hill, Liverpool L69 3GJ, UK; grjones@liv.ac.uk (G.R.J.); joe@liv.ac.uk (J.W.S.)

**Keywords:** contacts erosion, high-voltage circuit breaker (HVCB), optical-fibre based sensor, chromatic methodology, spectroscopic, online monitoring

## Abstract

Contact erosion is one of the most crucial factors affecting the electrical service lifetime of high-voltage circuit breakers (HVCBs). On-line monitoring the contacts’ erosion degree is increasingly in demand for the sake of condition based maintenance to guarantee the functional operation of HVCBs. A spectroscopic monitoring system has been designed based upon a commercial 245 kV/40 kA $SF_{6}$ live tank circuit breaker with copper–tungsten (28 wt % and 72 wt %) arcing contacts at atmospheric $SF_{6}$ pressure. Three optical-fibre based sensors are used to capture the time-resolved spectra of arcs. A novel approach using chromatic methods to process the time-resolved spectral signal has been proposed. The processed chromatic parameters have been interpreted to show that the time variation of spectral emission from the contact material and quenching gas are closely correlated to the mass loss and surface degradation of the plug arcing contact. The feasibility of applying this method to online monitoring of contact erosion is indicated.

## 1. Introduction

Contact erosion in high-voltage circuit breakers (HVCBs) is an inevitable consequence of current interruption and is caused primarily by vaporisation and splashing of molten material of the electrodes under the heating of arc plasma. The mass loss and surface structure changing of arcing contacts adversely affects the interruption performance of HVCBs and therefore determines their electrical service lifetime [1].

To avoid unexpected current interruption failure and save on maintenance costs, online monitoring of arcing contacts is increasingly in demand. So far, the monitoring of mass loss and maintenance of arcing contacts are mainly based on empirical knowledge or accumulations of breaking current and arcing time [2]. Over the past decades, many investigations have been undertaken to study the arcing contacts’ erosion. Generally, conclusions are drawn according to the energy balance at the arc–electrode interface without considering practical changes that might have occurred in the contact geometry and surface morphology [3,4,5,6,7].

Spectroscopic methods are widely used for arc plasma diagnostics [8,9]. Rouffet proposed a method to evaluate the temperature of thermal plasma based on the analysis of large spectral regions of plasma radiation. Hlina used a similar approach measuring the temperature of an air plasma cutting torch. Their approaches were based on rigorous scientific calculation of the net emission coefficient. The temperature was determined from the comparison of the calculated and measured values over a selected spectral band under controlled laboratory conditions.

The practical current breaking conditions in a CB are not fully understood. For instance, the local thermal equilibrium (LTE) condition, the optical thickness of radiation and the stability of the arc plasma column, etc. have not been verified. Therefore, there may be deficiencies in the predictions made with laboratory models for practical applications.

An aim of this investigation is to construct a spectroscopic online monitoring system using optical-fibre based sensors and seek a measurable parameter that has direct correlation with the mass loss of an arcing contact. It is not feasible to measure directly contact mass loss in an in service HVCB, but optical emissions from the arc plasma may contain relevant contact information and may be conveniently monitored. Such information is manifest in the spectra of such optical signals, so that an approach of correlating mass loss of an arcing contact with such arc spectra is proposed.

However, a flexible and effective data processing method is needed to extract useful information from the complex spectral emissions. Therefore, a chromatic method was selected for addressing the complex spectra. This method is a hierarchical approach used for dimensionality reduction, feature detection and cluster analysis, which is suitable for online condition monitoring [10,11,12]. It does not require complicated calculation. Instead, it is based on a comparison of different experimental data. When distinct patterns of the experimental data were derived, their correlation with a target measurand can be introduced through calibration. It offers the possibility for distinguishing changes in a system and for tracing the reasons for the changes without recourse to the need for detailed scientific calculations that may be suspect to noise interferences. Therefore, the applicability of chromatic methods can be extended to practical industrial conditions.

## 2. Experimental Test System

### 2.1. Circuit Breaker Unit

**Figure 1:**
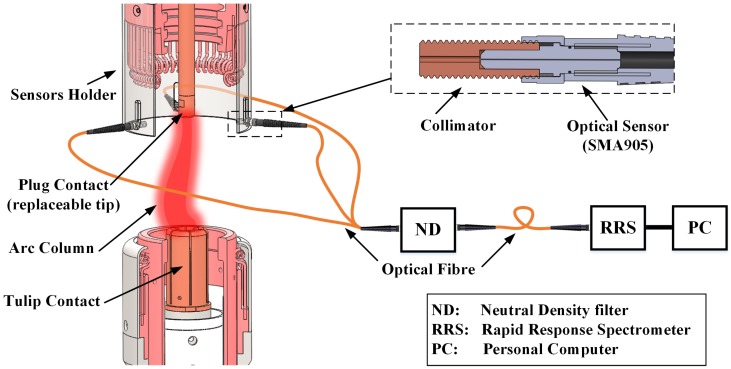
Schematic diagram of interrupter and optical measurement system.

A schematic diagram of the current interrupter unit used for the present tests is shown on Figure 1. This unit represented a commercial 245 kV/40 kA $SF_{6}$ live tank HVCB [13] operating at a pressure of 1bar. The material of the contacts was copper/tungsten (28 wt % of copper and 72 wt % of tungsten). The plug contact was attached to the top plate of the unit and its diameter was 18 mm. The contact tip was redesigned to be removable so that the mass loss could be measured by weighing before and after each operation with an electronic balance (Master${}^{pro}$ LP1200S, Sartorius, Göttingen, Germany) whose precision was 1 mg. The moving contact was connected to a driving mechanism for opening the contact gap whose maximum opening speed was 5 m/s. The fully opened gap was approximately 100 mm. The plug contact was used as the cathode and the tulip contact as the anode. Only the erosion of the plug contact was investigated. The main interrupter nozzle was removed to avoid the erosion process being affected by a possible interaction between the arc and the polytetrafluoroethylene (PTFE) nozzle material.

### 2.2. Optical Sensor System

The optical sensor system used for monitoring the spectral emission from an arc (Figure 1) was composed of three optical fibre based sensors that were installed at the same height as the plug contact surface and set at 120 degrees to each other. The observation area covered by each sensor was adjusted by a collimator to correspond to the contact diameter at the symmetry axis. Thus, the light emission from the arc could be captured equally by three sensors via a single channel rapid response spectrometer (Exemplar LS, B&W Tek, Newark, DE, USA, spectral resolution of 0.6 nm, minimum integration time 1050 μs, maximum data transfer speed 950 spectra per second via USB 3.0 cable). Approximately 10 spectra could be recorded during a half cycle arcing period of ∼10 ms. Since the absolute radiative energy from the arc was intense, before the optical light was fed into the spectrometer, an adjustable neutral density (ND) filter was used to avoid saturation and the raw data was compensated before processing. The spectral sensitivity of the spectrometer was corrected automatically before the raw data was transferred to the PC and the signal-to-noise ratio (SNR) was approximately 295 without on board averaging.

Additionally, the connection between the sensor and collimator needs to be carefully sealed to avoid potential damage caused by hot gas generated from the arc through gas circulation inside the collimator tube.

### 2.3. Power Test Circuit

**Figure 2:**
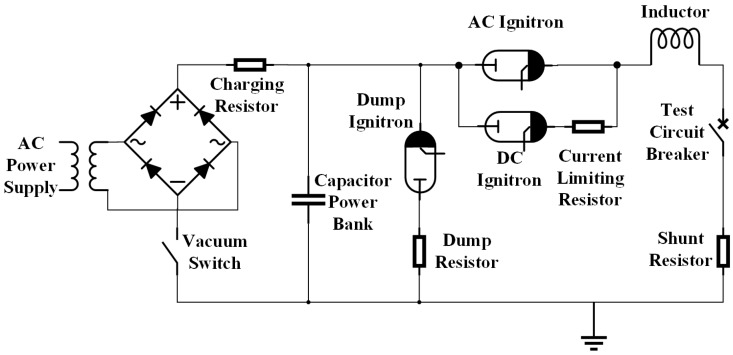
Schematic diagram of test circuit.

A capacitor/inductor resonance circuit (Figure 2) was used to generate a half cycle of alternating current. The main capacitor, used as the power source, had a capacitance of 35 mF, a maximum charging voltage of 6.3 kV and a maximum energy stored of 695 kJ. To provide a resonant current of 60 Hz, an inductance of 184 μH was connected in series with the capacitor bank. The capacitor charging system consisted of a transformer, rectifier, vacuum switch and charging resistor. A DC ignitron was installed to produce an arc initiation current through a current limiting resistor. This provided a low level direct current prior to an ignitron switching it to the AC current with an AC ignitron. A dump ignitron and resistor were installed to discharge the capacitor bank for safety reasons with precise timing as determined by the experiments. The current and voltage of the arc were measured with a shunt resistor of 1.19 mΩ and a high voltage probe (Tek P6015A, Tektronix, Beaverton, OR, USA), respectively. Trigger pulses required by the ignitrons were generated by the main control unit (MCU).

### 2.4. Test Procedures

**Figure 3:**
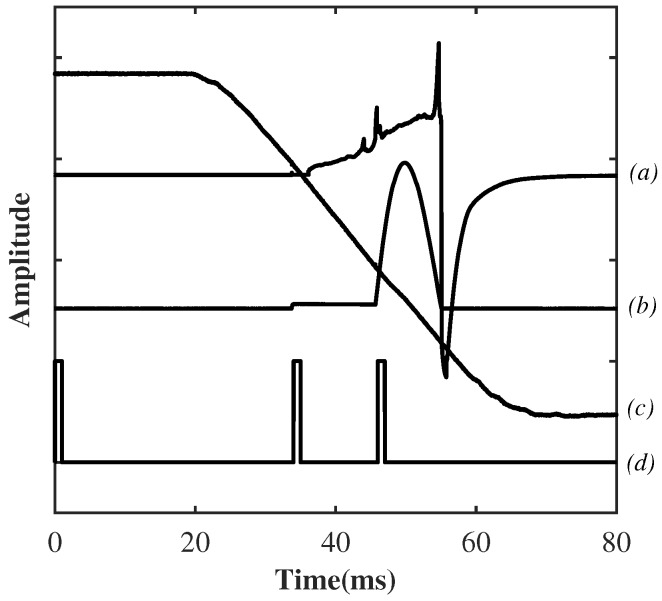
Experimental timing sequence and typical waveforms: (**a**) arc voltage; (**b**) arc current; (**c**) contact separation; and (**d**) trigger signals.

Some typical time varying waveforms for (a) arc voltage; (b) arc current; (c) contact travel and (d) trigger pulses are shown in Figure 3. A test was initiated by a “main trigger” at time zero. The main trigger was sent to the oscilloscope, spectrometer and also the circuit breaker operating mechanism. The tulip contact (Figure 1) started to move after approximately 20 ms after the initiation pulse (Figure 3). Thereafter, a second trigger pulse initiated a low level DC current (∼several hundred amperes) prior to the moment of contact separation in order to initialise the arcing and keep the gap between the two contacts conducting. After a fixed period of time, a third trigger was sent to initiate the positive half cycle of AC current lasted for from 46 ms until 56 ms (Figure 3). The displacement curve of the moving contact (Figure 3c) was also recorded using a linear position transducer. The timing sequence from test to test was highly repeatable.

Eight different peak current levels were used—namely, 5 kA${}_{p}$, 10 kA${}_{p}$, 15 kA${}_{p}$, 20 kA${}_{p}$, 25 kA${}_{p}$, 30 kA${}_{p}$, 35 kA${}_{p}$ and 40 kA${}_{p}$ (Table 1). For each current level, a set of five tests was performed for each set, starting with a new plug contact tip. The circuit breaker arcing chamber was opened after each single test so that the individual mass loss of the plug contact could be obtained. This enabled the relationship between contact mass loss and optical signature from the optical monitoring system to be studied. A unique test number was assigned to each of these tests as shown in Table 1.

## 3. Experimental Results

### 3.1. Mass Loss from Contact Weight Measurements

**Figure 4:**
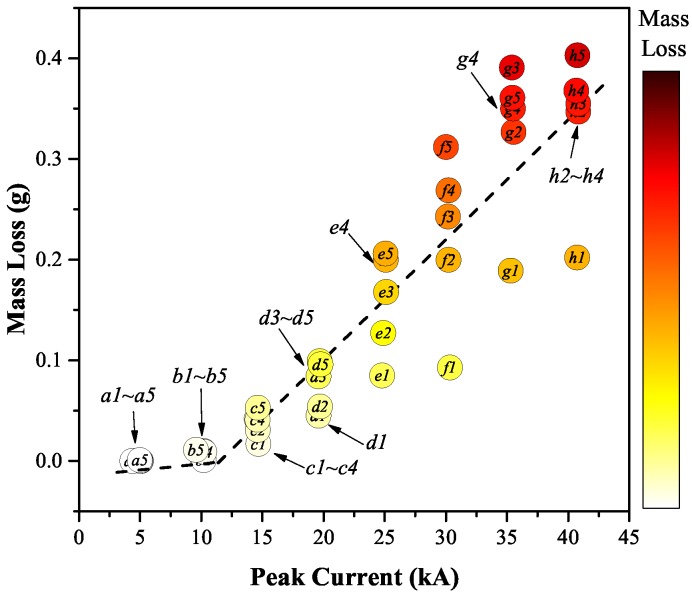
Measured mass loss of plug contact as a function of peak current.

Figure 4 shows the measured mass loss of the plug contact after each test as a function of peak current. The color bar shows the progression of increasing mass loss from white (bright) to red (dark). The test number is shown at the centre of each point.

The graph shows that the variation of the mass loss with peak current was nonlinear with an inflection point occurring between 10 kA to 15 kA. From 5 kA to 10 kA, the mass loss was relatively low because the absolute energy flux input into the contact was weak and the movement of the arc spot on the contact dissipated the energy at various locations along the contact surface [14]. Therefore, the energy available for heating and eroding the contact material was limited. From 15 kA up to 40 kA, a decrease in the arc spot mobility has been observed [14]. This produced more energy at selected fixed locations on the contact surface leading to increased temperatures and hence a higher rate of contact erosion. Such a discontinuous contact erosion rate as a function of increasing current has also been reported by other researchers [4,5,15].

For the same current levels, the mass loss rate generally increased with test numbers. This was especially so for the first test on a new contact, the mass loss being remarkably lower than those of subsequent tests. This effect was more pronounced at high current levels. The uncertainty of the prediction of mass loss is approximately ±110 mg when the current value is used as an indicator.

**Figure 5:**
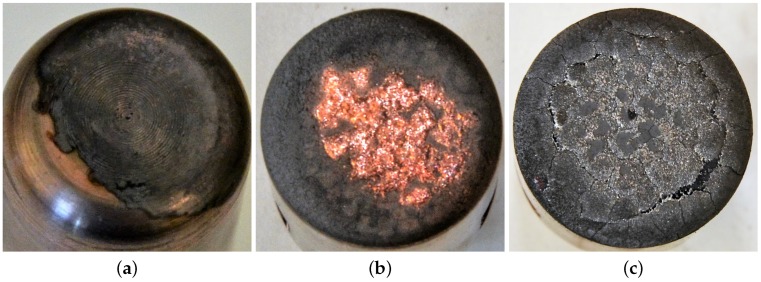
Contact surface images for different peak arc currents. (**a**) 5 kA, first test a1; (**b**) 25 kA, first test e1; and (**c**) 40 kA, fifth test h5.

Since the erosion rate of an individual breaking operation at “low” current levels is quite small compared to the accuracy of the mass loss measurements, only the test results over the 15 kA peak (inclusive) are considered. Apart from the mass loss, images of the plug contact tips were also taken (Figure 5) to provide more evidence about the mechanisms of contact erosion.

### 3.2. Time Varying Spectra

**Figure 6:**
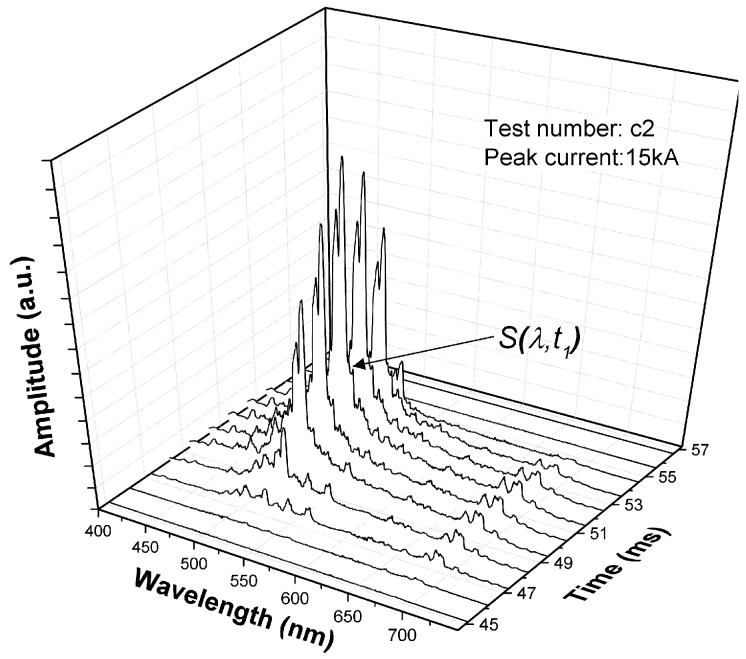
A typical time-resolved spectral signal (peak current 15 kA).

An example of a typical time varying spectrum is shown in Figure 6. Very strong emissions can be observed in the range from 500 nm to 550 nm. Emission lines from copper atom (Cu I), tungsten atom (W I) and sulphur ion (S II) are listed on Table 2. The spectra and their time variations are clearly complex.

It has been confirmed that electrode vapour dominates the arc when the current above 10 kA [17] and strong copper atom lines were observed (Figure 6). However, due to the self-absorption effect, the spectral lines were emerged with the strong continuum [17]. Conventionally, temperature and metal vapour density can be estimated via spectroscopic analysis when the arc plasma is proven to be LTE and optically thin [18,19,20]. Under the current breaking scenario, especially for currents above 10 kA, these assumptions are no longer valid. New approaches are needed to evaluate the state of the arc and its effect upon arcing contacts. The chromatic techniques described in Section 4.1 provides such a possibility.

## 4. Analysis of Test Results

### 4.1. Chromatic Techniques

**Figure 7:**
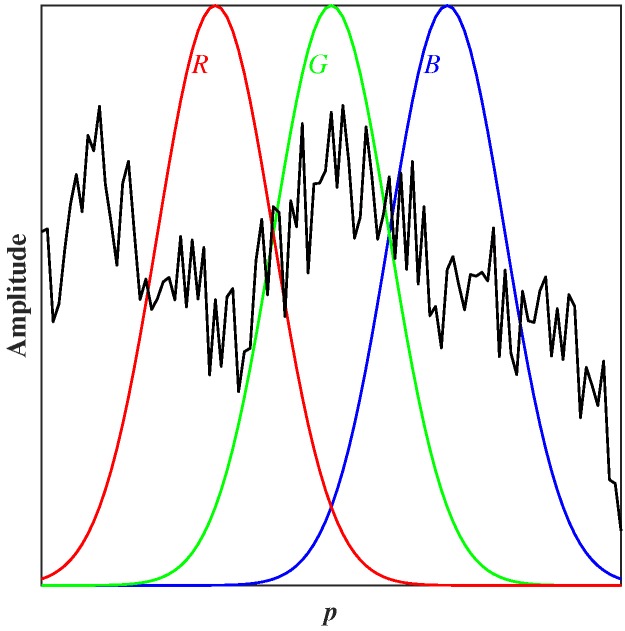
Schematic diagram of tri-stimulus chromatic processors (*R*, *G*, *B*) superimposed upon a complex signal S(p) as a function of a parameter *p*.

The complex, time varying, optical spectra (Figure 6) have been analysed using chromatic processing techniques. This approach involves addressing a complex signal with three non-orthogonal processors (*R*, *G*, *B*) (Figure 7) that are used to quantify various signal features such as the dominant wavelength (H), effective signal strength (L), equivalent signal spread (S) and relative magnitudes of three signal distribution components (*x*, *y*, *z*) [21].

With respect to a spectral signal, the chromatic method does not aim at dealing with specific wavelengths. It treats the components under each processor as an integral. It is worth noting that the overlapping between neighbour processors is compulsory [21]. However, optionally, in order to extract physically meaningful information, the positions and widths of the processors may be tuned according to each application.

The outputs of each processor are usually transformed into various chromatic parameters, e.g., HLS (i.e., hue, lightness and saturation) and xyz, to be interpreted easily. In this work, the xyz transformation was used, *x*, *y*, *z* being defined by the following equations [21]:

(1a) $$\begin{array}{c} x=R/(R+G+B) \end{array}$$

(1b) $$\begin{array}{c} y=G/(R+G+B) \end{array}$$

(1c) $$\begin{array}{c} z=B/(R+G+B) \end{array}$$

A signal may then be defined by its *x*, *y*, *z* coordinates as a single point on a two-dimensional *x*, *y*, *z* chromatic map [21]. Additionally, trends in signal changes may be represented by values of *x*, *y*, *z* as a function of factors producing the signal changes.

### 4.2. Primary Chromatic Analysis

**Figure 8:**
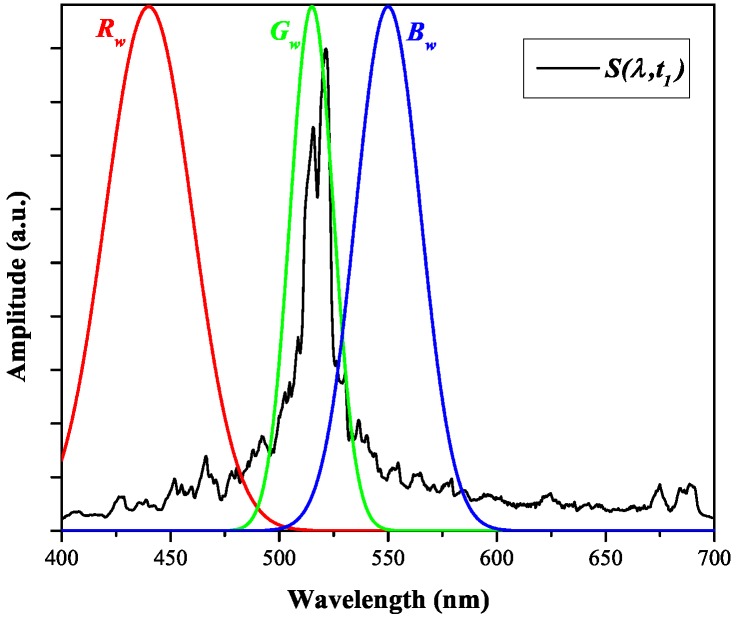
Wavelength domain processors $R_{w},G_{w},B_{w}$ superimposed upon a typical arc spectrum.

A primary chromatic analysis was undertaken of the arc spectral emission at the peak value ($t_{1}$, Figure 6) of each of the currents investigated (Table 1). An example of the deployment of three chromatic processors ($R_{w}$, $G_{w}$, $B_{w}$) for addressing such a wavelength spectrum is shown on Figure 8. The responses of the three processors covered the wavelength ranges occupied by the emission bands of tungsten atom (W I), copper atom (Cu I), and sulphur ion (S II) (Table 2). The outputs from the three processors ($R_{w}$, $G_{w}$, $B_{w}$) were converted into three primary chromatic parameters $x_{w}$($t_{1}$), $y_{w}$($t_{1}$) and $z_{w}$($t_{1}$) using Equation (1a–c).

**Figure 9:**
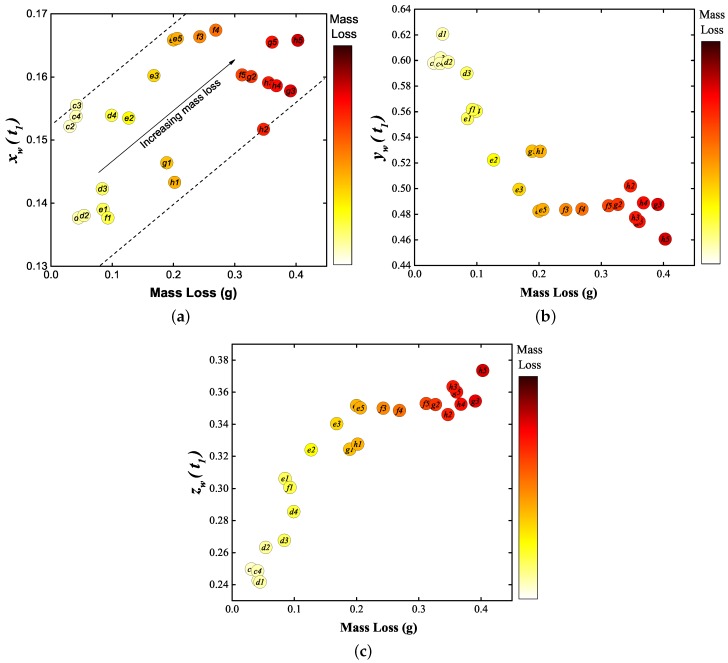
Primary chromatic parameters as functions of mass loss. (**a**) $x_{w}$($t_{1}$); (**b**) $y_{w}$($t_{1}$); and (**c**) $z_{w}$($t_{1}$).

Values of the primary chromatic parameters $x_{w}$($t_{1}$), $y_{w}$($t_{1}$) and $z_{w}$($t_{1}$) were plotted against the directly measured mass loss (Figure 4) to yield trend results shown on Figure 9a–c, respectively.

The uncertainty in the chromatic parameters values is mainly due to the spectrometer noise during recording. Since the SNR of the spectrometer is 295, combining with Equation (1a–c), the uncertainties of $x_{w}$($t_{1}$), $y_{w}$($t_{1}$) and $z_{w}$($t_{1}$) were estimated to be approximately ±0.0012. Considering that the variation of $x_{w}$($t_{1}$) is relatively small compared with $y_{w}$($t_{1}$) and $z_{w}$($t_{1}$), and only $y_{w}$(*t*) and $z_{w}$(*t*) were selected to be processed further as potential indicators of contact mass loss.

### 4.3. Secondary Chromatic Parameters

**Figure 10:**
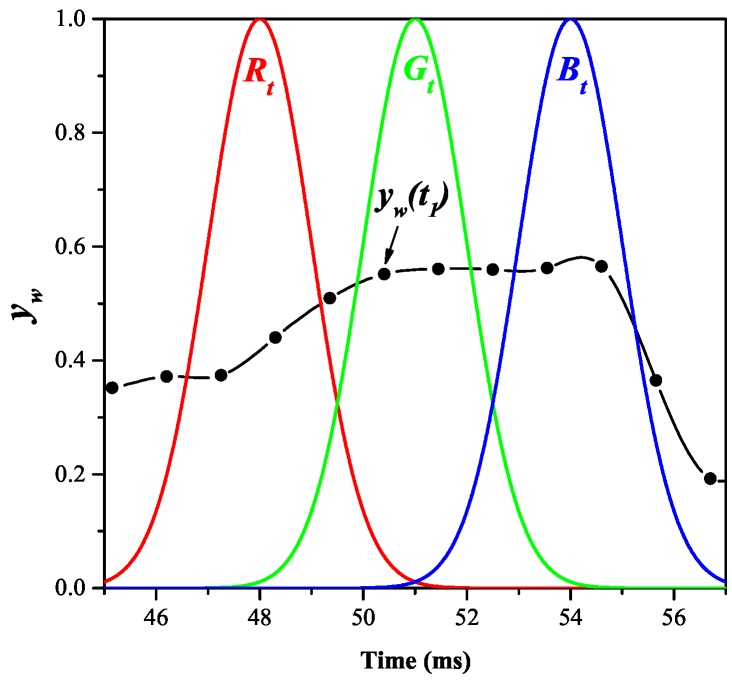
Example of time domain chromatic processors $R_{t},G_{t},B_{t}$ superimposed upon the time variation of wavelength chromatic parameter $y_{w}$ ($=G_{w0}/\left(R_{w0}+G_{w0}+B_{w0}\right)$).

Primary chromatic parameters at various times (*t*) during a current half cycle ($x_{w}\left(t\right)$, $y_{w}\left(t\right)$, $z_{w}\left(t\right)$) were calculated and the time variation of each during a half cycle was addressed by three time domain chromatic parameters ($R_{t}$, $G_{t}$, $B_{t}$). An example of the time variation of the primary chromatic (wavelength domain) parameter ($y_{w}\left(t\right)$) is shown in Figure 10 along with the three time domain chromatic processors superimposed. Nine secondary chromatic (time domain) parameters (e.g., $y_{tyw}$ and $y_{tzw}$, etc., Table 3) were evaluated from the $R_{t}$, $G_{t}$, and $B_{t}$ outputs.

**Figure 11:**
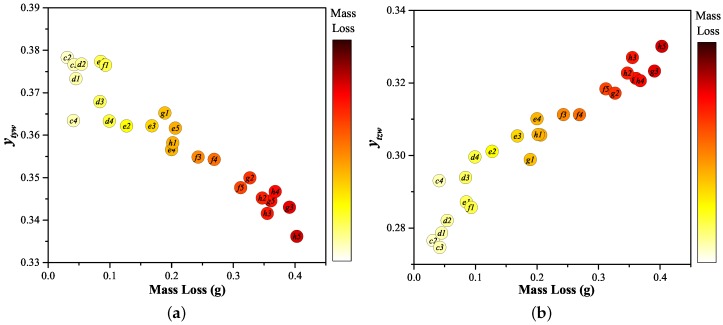
Secondary chromatic parameters as a function of mass loss. (**a**) $y_{tyw}$; and (**b**) $y_{tzw}$.

**Figure 12:**
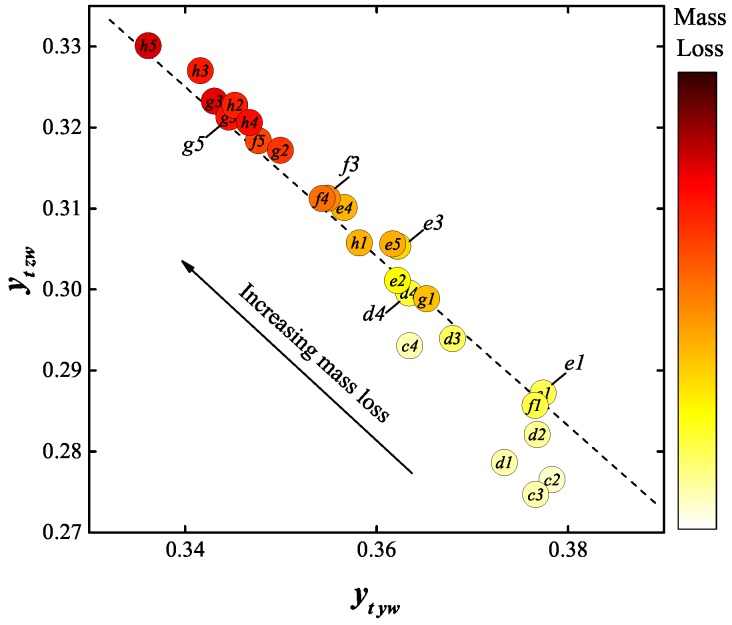
Secondary chromatic parameter map of $y_{tyw}$ vs. $y_{tzw}$.

The variation of each of two of these nine parameters ($y_{tyw}$ and $y_{tzw}$) with directly measured contact mass loss (Figure 4) is shown in Figure 11a,b. Figure 12 shows a secondary chromatic Map of $y_{tyw}$ versus $y_{tzw}$, from which a clear mass loss trend can be conveniently visualised.

## 5. Discussion

The primary chromatic parameters $x_{w}\left(t_{1}\right)$, $y_{w}\left(t_{1}\right)$, $z_{w}\left(t_{1}\right)$ shown in Figure 9a–c represent the relative emission from the W I, Cu I and S II bands, respectively, at $t_{1}$. It can be seen from Figure 9a, in general, that $x_{w}\left(t_{1}\right)$ increased slightly along with mass loss. Since the melting and vaporisation temperature of tungsten are much higher than the rest of elements involved in the arcing system, within the current range of this work (under 40 kA), $x_{w}\left(t_{1}\right)$ showed only a moderate increasing trend that is reasonable. Figure 9b shows that with increasing mass loss $y_{w}\left(t_{1}\right)$ generally decreased, indicating that the relative emission from Cu I band at $t_{1}$ became less pronounced. By contrast, $z_{w}\left(t_{1}\right)$ increased with mass loss indicating that the relative emission from S II (i.e., the surrounding gas) was becoming stronger. However, the relationships between each of these primary chromatic parameters and mass loss are not monotonic, particularly when the mass loss is higher than 0.2 g. The uncertainty of predicting mass loss using selected primary chromatic parameters is about ±93 mg. As such, there is a loss of sensitivity for determining mass loss level in this range, and recourse may be made to examine possibilities with secondary (time domain) chromatic processing.

Figure 11a,b shows that two selected secondary chromatic parameters $y_{tyw}$ and $y_{tzw}$ both varied monotonically with increasing contact mass loss and only moderate scatter. As such, and with further testing, they have a potential for being utilized for mass loss prediction.

The secondary parameter $y_{tyw}$ represents the magnitude during the medium time period (peak current) (i.e., $G_{t}$, Figure 10) relative to the other time periods of the relative emission from the Cu I band ($G_{w}$, Figure 8). The decrease in the value of $y_{tyw}$ with increasing mass loss may be explained as follows. Firstly, due to insufficient heating at the contact surface at “low” current levels, the emission peak from the CuI band coincided with the current peak, which occurred at the medium time period. Hence, the value of $y_{tyw}$ at low current levels tended to be greater. As the current increased, the emission peak shifted to the later time period because of continuous intensive heating from the arc. Therefore, the value of $z_{tyw}$ increased whilst that of $y_{tyw}$ decayed. Secondly, due to interaction between atoms and ions of copper with rising temperature, the intensity of atomic line has a non-monotonic dependence on temperature [22]. With rising temperature (i.e., rising mass loss), it first rises, before falling, after reaching a certain temperature. This is because at a certain temperature, there are more ions than atoms in the plasma. As copper ions’ emission lines (in the range over ∼600 nm) lie outside wavelength domain processors (i.e., $R_{w}$, $G_{w}$, $B_{w}$), the influences of copper ion emission upon chromatic parameters will be omitted in this work. By contrast, the medium time period relative emission from an S II band $y_{tzw}$ showed an opposite trend with increasing mass loss. This trend suggested the surrounding gas being heated so the emission from S II band ($B_{w}$, Figure 8) was more intense during the peak current phase with growing mass loss.

The combination of $y_{tyw}$ and $y_{tzw}$ offered an enhanced prediction of mass loss as shown in Figure 12. The absolute error of estimating mass loss with respect to current experimental conditions is approximately ±40 mg.

The measured erosion mass versus peak current results of Figure 4 shows the following:

- For peak currents <15 kA, the erosion is negligible and does not vary with repeated arcing (a1 → a2; b1 → b2, Figure 4).
- For peak currents >15 kA, the erosion level is not negible at the first test and increases with successive tests (e.g., 25 kA, e1 → e5, Figure 4).

The chromatically analysed test results show the same trend as the direct measurements (Figure 9 and Figure 11), whereas conventional estimates of the erosion level by integrating the arcing current with respect to time does not distinguish between the lower and higher currents’ behaviour.

Inspection of images of the contact after each successive test at a given peak current (Figure 5a–c suggests that the observed different behaviour at the lower (<15 kA) and higher (>15 kA) peak currents may be associated with different modes of contact wear.

At a low current of 5 kA, the contact surface did not suffer a major change (Figure 5a). However, after one test at a higher peak current of 25 kA, the contact surface was substantially changed (Figure 5b) with re-solidified copper (brown) from the sintered copper/tungsten of the contact material, indicating, due to the special properties of sintered copper/tungsten material [1], produced a layered structure. With an increase in peak current, more copper from deeper locations is melted and vaporized. After a few more tests on the same contact, especially for high current levels, the layered structure appeared to be formed with melted tungsten on the top, tungsten skeleton in the middle and copper/tungsten mixture at the bottom [23]. During the subsequent tests, the solid tungsten layer would be heated up to its melting temperature before the copper could be vaporized. This effect is consistent with the value of $y_{tyw}$ decreasing during subsequent tests since the formation of the “tungsten layer” delayed the appearance of the Cu I emission peak.

Thus, the processed secondary chromatic parameters are not only capable of predicting the mass loss of a contact (after calibration) but can also indicate the changes in the structure of the contact surface.

## 6. Conclusions

Investigations have reported about the erosion of a plug arcing contact in an HVCB subjected to a half cycle of current. Results of experiments with copper/tungsten arcing contacts subjected to a current of up to a 40 kA peak have been presented.

Mass loss from a contact following arcing have been measured for (a) a range of peak arc currents; and (b) repeated tests with the same contact and peak currents. The results of these tests show that there was a nonlinear relationship between the mass loss of the plug contact and a peak current with an inflection point occurring between 10 kA to 15 kA.

For each peak current level, the first test on a new contact tended to have a lower erosion rate than those of subsequent tests, and this effect was more pronounced at high current levels.

Time-resolved spectra of the contact eroding arcs have been obtained and processed using chromatic methods. The mass loss was correlated with selected secondary chromatic parameters $y_{tyw}$ and $y_{tzw}$ and the following conclusions were obtained:

- As mass loss increased, the ratio of the emission from Cu I band spectra around peak current decreased.
- The ratio of the emission from S II band spectra showed an opposite trend to Cu I, increasing around the peak current.

**Figure 13:**
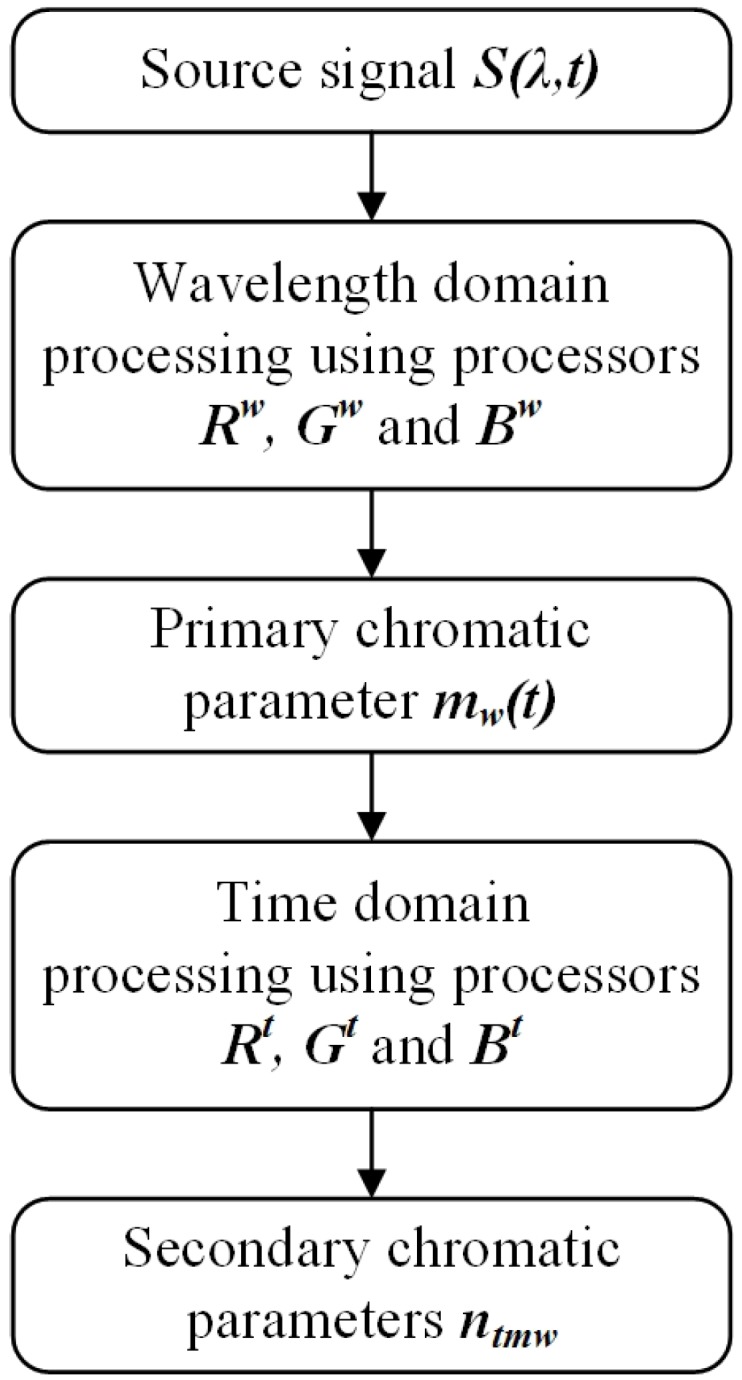
Flow chart for spectral data processing using chromatic methods (*m*, *n* are x,y,z).

The procedures followed to produce the chromatic results from the spectral data may be summarised by the flow chart given in Figure 13.

Images of plug contacts exposed to different currents have been presented which show various changes in the surface topology of a contact. Conclusions have been drawn about the relationship between the current effect on surface topology and the mass losses plus chromatic trends.

It may be possible to derive additional information from different secondary chromatic parameters using other transformation algorithms (refer to [21]). Moreover, the quantitative nature of the chromatic parameters can be used for online monitoring of contact erosion. The influence of arcing time, polarity of current and nozzle ablation on high current contact erosion, and its arcing spectral signatures require further investigation.

## Figures and Tables

**Table 1:** Assignment of test numbers.

Test Current	1st	2nd	3rd	4th	5th
5 kA	a1	a2	a3	a4	a5
10 kA	b1	b2	b3	b4	b5
15 kA	c1	c2	c3	c4	c5
20 kA	d1	d2	d3	d4	d5
25 kA	e1	e2	e3	e4	e5
30 kA	f1	f2	f3	f4	f5
35 kA	g1	g2	g3	g4	g5
40 kA	h1	h2	h3	h4	h5

**Table 2:** Strong emission lines of tungsten atom, copper atom and sulphur ion in a visible spectrum range [16].

Elements	Strong Emission Lines (nm)
W I	400.9, 407.4, 429.5, 430.2, 448.4, 465.9, 468.1, 484.4
Cu I	510.6, 515.3, 521.8
S II	542.9, 543.3, 545.4, 547.4, 551.0, 560.6, 564.0

**Table 3:** List of all secondary chromatic parameters.

Wavelength\Time	$x_{t}$	$y_{t}$	$z_{t}$
$x_{w}\left(t\right)$	$x_{txw}$	$y_{txw}$	$z_{txw}$
$y_{w}\left(t\right)$	$x_{tyw}$	$y_{tyw}$	$z_{tyw}$
$z_{w}\left(t\right)$	$x_{tzw}$	$y_{tzw}$	$z_{tzw}$
